# Effects of Pantothenic Acid Supplementation on Growth Performance, Carcass Traits, Plasma Parameters of Starter White Pekin Ducks Fed a Corn–Soybean Meal Diet

**DOI:** 10.3390/ani11102872

**Published:** 2021-09-30

**Authors:** Jing Tang, Yongbao Wu, Bo Zhang, Zhiguo Qi, Dawei Luo, Jian Hu, Wei Huang, Zhengkui Zhou, Ming Xie, Shuisheng Hou

**Affiliations:** 1 State Key Laboratory of Animal Nutrition, Key Laboratory of Animal (Poultry) Genetics Breeding and Reproduction, Ministry of Agriculture and Rural Affairs, Institute of Animal Sciences, Chinese Academy of Agricultural Sciences, Beijing 100193, China; tangjing198601@163.com (J.T.); woblin@163.com (Y.W.); zhangb950414@163.com (B.Z.); LDW17862513746@163.com (D.L.); tothewings@163.com (J.H.); hw-1115@163.com (W.H.); zhouzhengkui@caas.cn (Z.Z.); xieming01@caas.cn (M.X.); 2 Beijing General Station of Animal Husbandry, Beijing 100101, China; qzguo886@163.com; 3 Newhope Liuhe Group Co., Ltd., Qingdao 266000, China

**Keywords:** duck, pantothenic acid, requirement, growth performance, plasma parameter

## Abstract

**Simple Summary:**

The yield of meat duck has increased over the past decade. Precise nutrition of ducks would contribute to improve growth performance and feed efficiency. As one of the important B-vitamins, pantothenic acid is essential for animals, and the deficiency of this vitamin could lead to growth depression, high mortality, and abnormal glucose metabolism. Similarly, pantothenic acid is also needed for ducks. The objectives of this study were to evaluate the effects of dietary pantothenic acid levels on growth performance, carcass traits, and plasma biochemical parameters of ducks, as well as the pantothenic acid requirement of ducks based on conventional corn–soybean meal diets. The results showed that among all ducks, the birds fed the basal diet without pantothenic acid supplementation had the lowest growth performance, breast meat yield, and plasma pantothenic acid and glucose contents. In addition, all these parameters increased linearly or quadratically as the dietary pantothenic acid level increased. The pantothenic acid requirements of starter male white Pekin ducks were 13.29–15.0 mg/kg. The data potentially provides theoretical support for the utilization of pantothenic acid in duck production.

**Abstract:**

This study aimed to evaluate the effects of different dietary pantothenic acid levels on growth performance, carcass traits, and plasma biochemical parameters of starter Pekin ducks from 1 to 21 days of age, as well as the pantothenic acid requirement of starter ducks. A total of 384 one-day-old male white Pekin ducklings were assigned randomly into 6 dietary treatments, each with 8 replicate pens of 8 ducks. Ducks were fed conventional basal corn–soybean diets containing 8.5, 10.5, 12.5, 14.5, 16.5, and 18.5 mg/kg pantothenic acid for 21 days. Growth depression, poor pantothenic acid status, fasting hypoglycemia, and elevated plasma uric acid (UA) content were observed in the ducks fed the pantothenic acid-deficient basal diet (*p* < 0.05), and these adverse effects were ameliorated by pantothenic acid supplementation. Among all ducks, the birds fed the basal diet with no supplementation of pantothenic acid had the lowest body weight, average daily weight gain (ADG), average daily feed intake (ADFI), breast meat yield, and plasma pantothenic acid and glucose contents (*p* < 0.05), and the greatest plasma UA content (*p* < 0.05). In addition, all these parameters showed a linear or quadratic response as dietary pantothenic acid levels increased (*p* < 0.05). According to broken-line regression, the pantothenic acid requirements of starter male white Pekin ducks for body weight, ADG, and plasma pantothenic acid content were 13.36, 13.29, and 15.0 mg/kg, respectively. The data potentially provides theoretical support for the utilization of pantothenic acid in duck production.

## 1. Introduction

Pantothenic acid is a component of two coenzymes, coenzyme A and acyl-carrier-protein, which are involved in the metabolism of carbohydrates, lipids, and proteins [1,2]. Pantothenic acid deficiency could cause growth retardation, poor feathering, dermatosis, and high mortality in chicks, turkeys, and geese, and the requirements of this vitamin have been evaluated widely for these species [3,4,5,6,7,8,9]. Pantothenic acid is also essential for ducks and the deficiency of this vitamin could induce growth depression, exudate on eyelids, and high mortality in ducks [10,11,12]. The NRC [13] recommendation of pantothenic acid for white Pekin ducks is 11.0 mg/kg in either the starter or growing period, which refer to early literature [10]. Recently, we estimated pantothenic acid requirements of the modern strain of starter Pekin ducks based on the corn–soy isolate protein meal diets, ranging from 8.59 to 10.22 mg/kg [11]. However, in our previous study, these diets were not the typical diets for ducks. In commercial production of Pekin ducks, corn and soybean meal are still the most predominant ingredients in duck diets. In addition, pantothenic acid concentration in soybean meal (16.0 mg/kg) is much higher than that of soy protein isolate (4.2 mg/kg) [13]. In spite of this, corn–soybean meal diets are deficient in pantothenic acid for chicks [14]. Furthermore, the bioavailability of pantothenic acid varies in different feedstuff. It has been shown that pantothenic acid in corn and soybean meal is 100% bioavailable to chicks, whereas that in barley, wheat, and sorghum has about a 60% bioavailability [14]. Therefore, it is necessary to conduct a follow-up study to evaluate the pantothenic acid requirement of ducks on corn–soybean meal diets.

In addition, pantothenic acid deficiency induces abnormal glucose metabolism. It has been shown that pantothenic acid deficiency caused low fasting blood glucose levels and increased sensitivity to insulin in rats and dogs [15,16,17,18,19]. It is proposed that pantothenic acid appears to be part of a glucose carrier system [20]. Similarly, fasting hypoglycemia and decreased plasma insulin content were also observed in pantothenic-acid-deficient ducks based on corn–soy isolate protein meal basal diets [21]. However, whether pantothenic acid affects glucose metabolism in ducks is unknown based on corn–soybean meal diets. Therefore, the first objective of this study was to confirm the pantothenic acid requirement of ducks estimated in our previous study. Another objective of this study was to evaluate the effects of dietary pantothenic acid levels on the glucose metabolism of ducks.

## 2. Materials and Methods

All experimental procedures of the present study were performed strictly in accordance with the guidelines and were approved by the Animal Ethics Committee of Institute of Animal Sciences, Chinese Academy of Agricultural Sciences (Approval number: IASCAAS2019-19).

### 2.1. Experimental Design, Animals, and Housing

This study was conducted to evaluate the effects of different dietary pantothenic acid levels on growth performance, carcass traits, and plasma biochemical parameters of starter Pekin ducks from 1 to 21 days of age, as well as the pantothenic acid requirement of starter ducks. A total of 384 one-day-old male white Pekin ducklings (from Pekin duck breeding center in the Chinese Academy of Agricultural Sciences) were assigned randomly into 6 dietary treatments, each with 8 replicate pens of 8 ducks. Each replicate pen had a similar bird body weight. The birds were housed in plastic-wire-floored pens (length 2 m × width 0.75 m × height 0.4 m) and had free access to pelleted feed and water. The initial temperature of 30 °C was kept for 3 days and then gradually reduced to 22 °C thereafter until 21 days of age. The lighting was kept continuous (24 h) throughout the whole study.

### 2.2. Diets

The experimental diets were formulated to meet or exceed the recommendations for starter ducks provided by Ministry of Agriculture of China (2012) [22] for all nutrients except for pantothenic acid. A pantothenic acid-deficient basal diet was formulated with corn and soybean (Table 1). The corn–soybean basal diet was prepared as mash and then supplemented with 0, 2, 4, 6, 8, or 10 mg pantothenic acid/kg diet as calcium pantothenate (99% purity, Hangzhou Xinfu Technology Co. Ltd., Zhejiang, China) to produce 6 experimental diets. All diets were cold-pelleted at the room temperature. The pantothenic acid content of the basal diet was 8.5 mg/kg, analyzed by high-performance liquid chromatography (HPLC; Agilent 1290, Agilent Technologies) coupled with triple-quadrupole mass spectrometry (LC/MS; Agilent 6470, Agilent Technologies). The calculated total pantothenic acid contents of the 6 experimental diets were 8.5, 10.5, 12.5, 14.5, 16.5, and 18.5 mg/kg, respectively.

### 2.3. Sample Preparation and Data Collection

At 14 and 21 days of age, all the ducks were fasted overnight, then the body weight and diet consumption of the ducks from each pen were measured. The average daily weight gain (ADG), average daily feed intake (ADFI), and feed conversion ratio (FCR) were calculated for birds at different stages (1 to 14 days, and 1 to 21 days). At 21 days of age, after fasting overnight, two ducks close to the pen, of average body weight, were selected from each replicate pen and bled through a wing vein. Blood was collected into heparin sodium-containing tubes and plasma was separated, then stored at −20 °C. These birds were then slaughtered, and the breast meat, leg meat, and abdominal fat were separated and weighed. The percentage yield for the breast meat, leg meat, and abdominal fat were expressed as relative weight to live body weight.

### 2.4. Measurements

The plasma activities of plasma alanine transaminase (ALT) and aspartate transaminase (AST), and plasma concentrations of total protein (TP), albumin (ALB), glucose, and uric acid (UA) were measured based on a spectrophotometric method using an automatic analyzer (Hitachi 7080, Tokyo, Japan) with corresponding kits (Maccura, Chengdu, China) based on their specifications.

Feed and plasma pantothenic acid concentrations were measured by HPLC (Agilent 1290) coupled with triple-quadrupole mass spectrometry (Agilent 6470) according to the methods described previously [23]. Prior to LC/MS analysis, feed and plasma samples were prepared according to the methods described previously [24,25]. The peak was identified by the pure authentic standards (Sigma-Aldrich, St. Louis, MO, USA).

### 2.5. Statistical Analyses

The data was analyzed by ANOVA using the general linear model (GLM) procedure of SAS software (SAS Inst. Inc., Cary, NC, USA) with the pen being the experimental unit. A significant difference among all dietary treatments was determined at *p* < 0.05 by Turkey test. Variability in the data is expressed as the standard error of the means (SEM). The dose–response effect of supplemental pantothenic acid was computed using the polynomial comparison for linear and quadratic effects. The pantothenic acid requirements for ducks were estimated for the broken-line model by using the nonlinear regression analysis (NLIN) of SAS as described previously [26].

## 3. Results

### 3.1. Growth Performance and Carcass Traits

As is shown in Table 2 and Table 3, the ducks fed the basal diet without pantothenic acid supplementation had the lowest body weight, ADG, and ADFI from hatch to 14 days of age (*p* < 0.05, Table 2) or from hatch to 21 days of age (*p* < 0.05, Table 3) among all ducks. As the dietary pantothenic acid increased, all these parameters increased linearly (*p* < 0.05, Table 2; *p* < 0.05, Table 3) and reached a plateau when the dietary pantothenic acid was above 12.5 mg/kg. The dietary pantothenic acid levels did not affect FCR of ducks during the experimental period (*p* > 0.05, Table 2; *p* > 0.05, Table 3).

As is shown in Table 4, dietary pantothenic acid levels affected breast meat yield (*p* < 0.05), but not the yield of leg meat and abdominal fat (*p* > 0.05). The ducks fed the basal diet with no supplementation of pantothenic acid had the lowest percentage of breast meat yield among all birds (*p* < 0.05), and it increased quadratically as dietary pantothenic acid increased (*p* < 0.05).

### 3.2. Plasma Parameters

The effects of dietary pantothenic acid levels on plasma biochemical parameters of starter white Pekin ducks are shown in Table 5. Dietary pantothenic acid levels did not affect plasma ALT, AST, TP, and ALB (*p* > 0.05). The ducks fed the basal diet without pantothenic acid supplementation had the lowest plasma pantothenic acid and glucose contents, but the greatest plasma UA content among all birds (*p* < 0.05). Plasma pantothenic acid and glucose contents increased while plasma UA content decreased as dietary pantothenic acid increased (*p* < 0.05).

### 3.3. Pantothenic Acid Requirement of Ducks

The pantothenic acid requirements of starter ducks according to the broken-line regression are shown in Table 6. Based on this regression, the pantothenic acid requirements of starter white Pekin ducks for body weight, ADG, and plasma pantothenic acid content were 13.36, 13.29, and 15.0 mg/kg, respectively.

## 4. Discussion

### 4.1. Growth Performance and Carcass Traits

In contrast to a purified diet or the corn and soy protein isolate basal diet used in previous studies [10,11], a corn and soybean basal diet was employed in the present study. The characteristic symptoms of pantothenic acid-deficient ducks reported by previous studies [10,11], such as poor feathering, dermatosis, excessive exudate from the eyes, and high mortality, were not observed in this study. The discrepancy between the present and previous results could be due to the difference in the basal diets; the pantothenic acid content of the basal diet in this study (8.5 mg/kg) was remarkably greater than that in previous study (4.65 mg/kg) [11], which may cause less severe pantothenic acid deficiency. In the present study, the ducks fed the corn–soybean meal basal diet without pantothenic acid supplementation had the worst growth performance and breast meat yield, but these negative effects can be alleviated by pantothenic acid supplementation, which is in line with the results in chicks, turkey poults, geese, and ducks [3,4,5,6,7,8,9,10,11,12]. Therefore, the results above suggest that a corn–soybean meal-based diet is deficient in pantothenic acid for ducks, and it is necessary to supplement pantothenic acid to diet. Furthermore, ducks fed a corn–soybean meal basal diet without pantothenic acid supplementation had a slightly lower growth performance than those birds with pantothenic acid supplementation in the present study, whereas the birds fed a corn–soy protein isolate basal diet had a dramatic decline in growth performance [11]. Our finding is consistent with the results in chicks, showing only a slight improvement in performance when 10 mg pantothenic acid/kg was added to a corn–soybean basal diet [14].

### 4.2. Plasma Parameters

Plasma or tissue pantothenic acid concentration could be a useful biomarker for pantothenic acid status. It has been shown that tissue pantothenic acid markedly decreased in pantothenic-acid-deficient shrimp, fish, and ducks [11,12,21,27,28,29]. In line with these previous studies, the ducks fed the basal diet without pantothenic acid supplementation had the lowest plasma pantothenic acid concentration among all ducks in the present study, which also showed the poorest growth and breast meat yield. However, these adverse effects could be eliminated by increasing dietary pantothenic acid levels. In agreement with studies in shrimp [29] and fish [27,28], the plasma pantothenic acid concentration increased linearly as the dietary pantothenic acid level increased in the present study, which was also accompanied with the simultaneous improvement of growth performance and carcass traits.

Pantothenic acid deficiency leads to abnormal glucose metabolism. In the present study, pantothenic acid deficiency caused fasting hypoglycemia and elevated UA content, confirming our previous results [21]. This finding is also consistent with previous studies in rats and dogs, showing pantothenic acid deficiency caused by low fasting blood glucose levels and increased sensitivity to insulin [15,16,17,18,19]. Recently, we found that pantothenic acid deficiency caused abnormal glucose absorption in ducks, indicated by a decreased expression of intestinal glucose transporter 2 (GLUT2), which may lead to hypoglycemia [21]. This explanation is supported by the previous finding that pantothenic acid appears to be part of a glucose carrier system [20]. In addition, pantothenic acid deficiency elevated the plasma UA content in ducks in the present study, indicating reduced excretion via the kidneys. Uric Acid is an oxidation end product of purine metabolism. This finding is consistent with previous studies in mammals showing that pantothenic acid deficiency imposes stress on the adrenal cortex, resulting in exhaustion of the gland and adrenal hypofunction [17,18].

### 4.3. Pantothenic Acid Requirement of Ducks

The broken-line regression has been extensively employed to evaluate the pantothenic acid requirements for ducks [10,11,12], chicks [7], shrimp [29], and fish [27,28,30]. Furthermore, the body weight, ADG, or plasma pantothenic acid content of ducks showed a linear response to increasing dietary pantothenic acid levels. Therefore, this regression could be used to evaluate the pantothenic acid requirements of starter ducks in this study. Based on broken-line regression, the pantothenic acid requirements of starter male white In this study, Pekin ducks for body weight, ADG, and tissue pantothenic acid content were 13.36, 13.29, and 15.0 mg/kg, respectively, and greater than 10.04, 10.05, and 10.22 mg/kg as reported by Tang et al. (2020a) [11] or 11 mg/kg as reported by Hegsted and Perry (1948) [10]. The differences between the present and previous results may be due to the different pantothenic acid contents in the basal diets (8.5 mg/kg versus 4.65 mg/kg). Furthermore, the pantothenic acid requirements estimated for tissue saturation (15.0 mg/kg) were greater than those for growth performance (13.36 and 13.29 mg/kg) in the present study, which is in line with the findings in fish [27,28] and ducks [11,12]. One explanation is that the tissue pantothenic acid concentration responds more rapidly to dietary pantothenic acid intake than growth performance [28].

## 5. Conclusions

The ducks fed the corn–soybean meal basal diet without pantothenic acid supplementation showed the depressed growth performance and breast meat yield of starter Pekin ducks, as well as fasting hypoglycemia, but these adverse effects can be reversed by pantothenic acid supplementation. According to broken-line regression, the pantothenic acid requirements for the modern breed of male white Pekin ducks from hatch to 21 days of age based on the corn–soybean basal diet for body weight, ADG, and plasma pantothenic acid content were 13.36, 13.29, and 15.0 mg/kg, respectively. The data potentially provides theoretical support for the utilization of pantothenic acid in duck production.

## Figures and Tables

**Table 1:** Composition of the pantothenic acid-deficient basal diet from hatch to 21 days of age (g/kg as-fed).

Item	Value
Ingredient, g/kg	
Corn	636
Soybean	324
Limestone	8
Dicalcium phosphate	16
Vitamin and trace mineral premix ^a^	10
Sodium chloride	3
DL-Methionine	2
L-Lysine·HCl	1
Total	1000
Calculated composition, g/kg	
Metabolizable energy ^b^, kcal/kg	2900
Crude protein	200.3
Calcium	9.6
Nonphytate phosphorus	4.2
Lysine	11.1
Methionine	5.1
Methionine + cysteine	8.5
Threonine	8.1
Tryptophan	2.5
Arginine	12.8
Pantothenic acid ^c^, mg/kg	8.5

^a^ Supplied per kilogram of total diet: Cu (CuSO_4_•5H_2_O), 10 mg; Fe (FeSO_4_•7H_2_O), 60 mg; Zn (ZnO), 60 mg; Mn (MnSO_4_•H_2_O), 80 mg; Se (NaSeO_3_), 0.3 mg; I (KI), 0.2 mg; choline chloride, 1000 mg; vitamin A (retinyl acetate), 10,000 IU; vitamin D_3_ (Cholcalciferol), 3000 IU; vitamin E (DL-α-tocopheryl acetate), 20 IU; vitamin K_3_ (menadione sodium bisulfate), 2 mg; thiamin (thiamin mononitrate), 2 mg; riboflavin, 10 mg; pyridoxine hydrochloride, 4 mg; cobalamin, 0.02 mg; nicotinic acid, 50 mg; folic acid, 1 mg; biotin, 0.2 mg. ^b^ The value is calculated according to the AME of ducks (Ministry of Agriculture of China, 2012). ^c^ The value was analyzed by high-performance liquid chromatography coupled with triple-quadrupole mass spectrometry.

**Table 2:** Effect of dietary pantothenic acid levels on growth performance of male white Pekin ducks from hatch to 14 days of age ^1^.

Dietary Pantothenic Acid (mg/kg)	Body Weight (g)	ADG (g/d/Bird)	ADFI (g/d/Bird)	FCR (g/g)
8.5	579 ^c^	37.5 ^c^	55.2 ^b^	1.47
10.5	588 ^bc^	38.1 ^bc^	55.6 ^b^	1.46
12.5	602 ^ab^	39.1 ^ab^	57.3 ^ab^	1.47
14.5	601 ^ab^	39.0 ^ab^	57.0 ^ab^	1.46
16.5	611 ^a^	39.7 ^a^	59.2 ^a^	1.49
18.5	602 ^ab^	39.1 ^ab^	58.3 ^a^	1.49
SEM	2.94	0.21	0.36	0.01
*p*-value				
Pantothenic acid	0.016	0.017	0.004	0.285
Pantothenic acid linear	0.002	0.002	<0.001	0.096
Pantothenic acid quadratic	0.089	0.084	0.522	0.153

ADG: average daily weight gain; ADFI: average daily feed intake; FCR: feed conversion ratio; SEM: standard error of the mean. ^1^ Results are the means with *n* = 8 per treatment. ^a–^^c^ Means with different superscripts within the same column differ significantly (*p* < 0.05).

**Table 3:** Effect of dietary pantothenic acid levels on growth performance of male white Pekin ducks from hatch to 21 days of age ^1^.

Dietary Pantothenic Acid (mg/kg)	Body Weight (g)	ADG (g/d/Bird)	ADFI (g/d/Bird)	FCR (g/g)
8.5	1155 ^b^	52.4 ^b^	91.9 ^b^	1.75
10.5	1158 ^b^	52.5 ^b^	91.9 ^b^	1.75
12.5	1180 ^ab^	53.6 ^ab^	94.0 ^ab^	1.75
14.5	1173 ^ab^	53.3 ^ab^	93.4 ^b^	1.76
16.5	1194 ^a^	54.3 ^a^	97.1 ^a^	1.79
18.5	1190 ^a^	54.1 ^a^	97.0 ^a^	1.79
SEM	4.39	0.21	0.55	0.01
*p*-value				
Pantothenic acid	0.027	0.028	0.004	0.073
Pantothenic acid linear	0.006	0.006	<0.001	0.015
Pantothenic acid quadratic	0.526	0.511	0.849	0.309

ADG: average daily weight gain; ADFI: average daily feed intake; FCR: feed conversion ratio; and SEM: standard error of the mean.^1^ Results are means with *n* = 8 per treatment. ^a,^^b^ Means with different superscripts within the same column differ significantly (*p* < 0.05).

**Table 4:** Effect of dietary pantothenic acid levels on carcass traits of the 21-day-old male white Pekin ducks ^1,2^.

Dietary Pantothenic Acid (mg/kg)	Breast Meat	Leg Meat	Abdominal Fat
8.5	1.91 ^b^	9.86	0.74
10.5	2.18 ^a^	9.76	0.80
12.5	2.17 ^a^	10.0	0.85
14.5	2.09 ^a^	9.81	0.87
16.5	2.16 ^a^	9.96	0.85
18.5	2.02 ^ab^	9.88	0.78
SEM	0.026	0.75	0.16
*p*-value			
Pantothenic acid	0.019	0.937	0.147
Pantothenic acid linear	0.446	0.746	0.213
Pantothenic acid quadratic	0.004	0.848	0.016

SEM: standard error of the mean.^1^ Results are the means of 8 replicates of 2 ducks each. ^2^ The percentage yield is calculated using the following equation: Yield = (breast meat, leg meat, or abdominal fat weight) × 100%/live body weight. ^a,b^ Means with different superscripts within the same column differ significantly (*p* < 0.05).

**Table 5:** Effect of dietary pantothenic acid levels on plasma biochemical parameters of the 21-day-old male white Pekin ducks ^1^.

Dietary Pantothenic Acid (mg/kg)	ALT (U/L)	AST (U/L)	TP (g/L)	ALB (g/L)	Glucose (mmol/L)	UA (μmol/L)	Pantothenic Acid (nmol/L)
8.5	34.1	14.8	17.1	0.98	8.94 ^c^	117 ^a^	500 ^b^
10.5	34.7	16.4	16.4	1.02	9.30 ^b^	92.5 ^b^	509 ^b^
12.5	34.1	14.3	16.6	0.98	9.48 ^ab^	92.2 ^b^	715 ^ab^
14.5	37.3	14.7	17.2	1.01	9.81 ^a^	93.4 ^b^	768 ^a^
16.5	41.3	15.0	16.9	0.99	9.51 ^ab^	98.3 ^b^	723 ^ab^
18.5	38.8	17.0	17.3	1.01	9.25 ^bc^	102 ^ab^	870 ^a^
SEM	1.49	0.54	0.14	0.07	0.06	2.61	38.7
*p*-value							
Pantothenic acid	0.675	0.637	0.332	0.397	<0.001	0.046	0.011
Pantothenic acid linear	0.129	0.506	0.225	0.533	0.020	0.423	<0.001
Pantothenic acid quadratic	0.932	0.339	0.230	0.650	<0.001	0.007	0.653

ALT: alanine transaminase; AST: aspartate transaminase; TP: total protein; ALB: albumin; UA: uric acid; SEM: standard error of the mean.^1^ Results are the means of 8 replicates of 2 ducks each. ^a–c^ Means with different superscripts within the same column differ significantly (*p* < 0.05).

**Table 6:** Pantothenic acid requirements of male white Pekin ducks from hatch to 21 days of age based on broken-line regression analysis.

Response Criterion	Regression	Requirement (mg/kg)	95% Confidence Interval (mg/kg)	*p*-Value	R^2^
Body weight	*y* = 1182 – 6.33 × (13.36 – *x*)	13.36	9.21 to 17.50	0.025	0.848
ADG	*y* = 53.69 – 0.31 × (13.29 − *x*)	13.29	9.25 to 17.33	0.025	0.850
Plasma pantothenic acid	*y* = 796.3 – 50.11 × (15.0 – *x*)	15.0	11.28 to 18.73	0.002	0.840

ADG: average daily gain.

## Data Availability

Not applicable.
